# Effects of Rosin Powder Application on the Frictional Behavior Between a Finger Pad and Baseball

**DOI:** 10.3389/fspor.2020.00030

**Published:** 2020-04-08

**Authors:** Takeshi Yamaguchi, Naoto Yamakura, Shinnosuke Murata, Takehiro Fukuda, Daiki Nasu

**Affiliations:** ^1^Department of Finemechanics, Graduate School of Engineering, Tohoku University, Sendai, Japan; ^2^Graduate School of Biomedical Engineering, Tohoku University, Sendai, Japan; ^3^NTT Communication Science Laboratories, Nippon Telegraph and Telephone Corporation, Atsugi, Japan

**Keywords:** baseball pitching, friction, finger, powder, grip

## Abstract

Rosin powder, which is composed of magnesium carbonate powder and pine resin, is often used as a grip-enhancing agent in baseball pitching. However, the effect of rosin powder on friction at the baseball–human finger interface remains unclear. This study aimed to investigate the effect of rosin powder on the friction coefficient between a baseball and a finger using sliding friction tests. Ten young adult males participated in this study who were asked to slide the index finger of their dominant hand over the leather skin of a baseball adhered to the force sensor, which was not a real baseball pitching situation. Our findings suggest that rosin powder application stabilizes friction under both dry and wet conditions; that is there was less dependence of the friction coefficient on the normal force and less variation in the friction coefficient among individuals. For most participants, the friction coefficient was not necessarily increased by the presence of rosin powder at the finger pad–leather sheet interface under dry conditions. However, under wet conditions, rosin powder application increased the friction coefficient compared with the non-powdered condition in the large normal force condition, indicating the efficacy of rosin powder as a grip-enhancing agent.

## Introduction

The control of slippage between a hand and an object is fundamental to improving athletic performance in sports that require a strong grip, such as baseball and rock-climbing. The main issue in controlling slippage is mitigating the change in friction caused by water or perspiration. To prevent slippage between a hand and an object, athletes have been known to use gloves, wipe water, and perspiration from their hands, and use grip-enhancing powders. Among these methods, applying powder seems to be the most effective option for maintaining grip under both dry and wet conditions. Magnesium carbonate powder and rosin powder, which is mainly composed of magnesium carbonate powder and pine resin, are common grip-enhancing powders used in sports.

Magnesium carbonate powder, i.e., “chalk,” is believed to help absorb perspiration and improve grip on the item being held onto while climbing (Li et al., 2001; Fuss et al., 2004; Fuss and Neigl, 2006; Bourne et al., 2011; Amca et al., 2012; Kilgas et al., 2016). Li et al. (2001) investigated the effect of magnesium carbonate powder on the sliding friction coefficient at the fingertip–rock surface interface and found that the coefficient decreased under both dry and wet conditions. Fuss et al. (2004) and Fuss and Neigl (2006) also found that magnesium carbonate powder use reduced the friction coefficient compared with no grip agent use on the hands for holding stones. Kilgas et al. (2016) reported that there were no differences in the friction coefficient of hands with and without the use of magnesium carbonate powder. These findings are contradictory to the supposed role of the powder, which is empirically known as a grip-enhancing agent. Conversely, Amca et al. (2012) reported a positive effect of using rosin powder on the friction coefficient between the fingers of climbers and two different rock types (sandstone and limestone), i.e., an increased friction coefficient was observed. Carré et al. (2012) investigated the effects of magnesium carbonate powder and rosin powder on the sliding friction between the fingers and a polished steel surface and showed that the powder decreased the friction coefficient compared with no application of the powder under dry condition; however, the powder increased the friction coefficient when the fingers were wet. Yamaguchi and Hokkirigawa (2015) conducted a sliding control test to investigate the controllability of sliding a vertically grasped cylindrical bar with and without the use of magnesium carbonate or rosin powder. Their results indicated that both powders reduced the degree of variation in the sliding velocity of the grasped bar and improved the controllability of its sliding motion compared with dry and wet non-powdered conditions. These authors also pointed out that powder application decreased the range of variation in the friction force rather than increasing the magnitude of frictional force between a rubber-gloved hand and the grasped bar during sliding under dry and wet conditions. As described, controversies remain regarding the efficacy of powders used as grip-enhancing agents.

The literature indicates the positive and negative effects of friction between finger and object in throwing sports such as rugby and Frisbee (Tomlinson et al., 2009; Lewis et al., 2014). While throwing a rugby ball, high friction is required to spin the ball about its long axis in addition to catching and holding the ball, and high friction between finger and rugby ball is necessary for an accurate pass. In contrast, in Frisbee, high friction with the use of gloves decreased the accuracy of catching and passing the Frisbee. Although friction between finger and object is thought to be important in throwing sports, as demonstrated in the studies cited, no researchers have investigated the effects of grip-enhancing powder application to fingers on throwing performance.

In baseball pitching, rosin powder is often applied between the finger pad and a baseball. This powder is empirically considered a grip-enhancing agent; however, whether rosin powder actually increases the friction between the finger pad and the ball remains unclear. In four-seam fastball pitching, the ball begins to roll up to the tip of the finger ~10 ms before ball release (Matsuo et al., 2018). During this period, shear (friction) force between finger pads and the baseball may affect the rotational speed and ball direction. Thus, understanding the frictional characteristics of the finger pads and ball is important. Kinoshita et al. (2017) measured the resultant and shear forces imparted by fingers onto a baseball during four-seam fastball pitching and found that a proportional relationship existed between finger force and ball speed. From their results, the approximated normal force applied by the index finger and middle finger increases after stride foot contact and exhibits a bimodal profile, with initial and second peaks at 40 ms and 6–7 ms before ball release, respectively. Then, the normal force reduces from 80 N (second peak of the normal force) to 0 N at the instant of ball release when the ball velocity is 31.4 m/s. The effect of normal load on the friction coefficient of human skin is significant as a result of contact pressure–dependence of friction coefficient (Derler et al., 2007, 2009); thus, sliding friction testing between a finger and baseball material should be performed under a wide range of normal force conditions.

To elucidate the effect of rosin powder applied to the finger pad in baseball pitching, we investigated the impact of rosin powder application on the friction coefficient between a sheet of baseball leather and the human finger under dry and wet conditions. The normal force between a finger pad and a baseball varies widely during the ball-releasing process (Kinoshita et al., 2017); therefore, we investigated the friction coefficient under a wide range of normal force conditions between the finger pad and the leather baseball sheet surface.

## Materials and Methods

### Experimental Procedure

**Figure 1:**
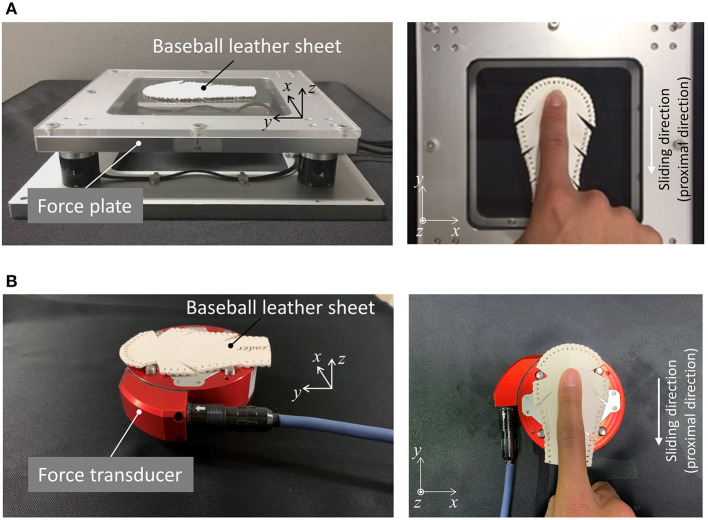
Experimental setup **(A)** for the low normal force condition (*F*_z_ ≤ 10 N) and **(B)** for large normal force condition (*F*_z_ > 10 N).

The sliding friction test between human index fingers and the baseball leather sheet was performed using a force plate (Figure 1A) (Shokkaku force plate; Tec Gihan Co., Ltd., Japan) and a capacitive six-axis force sensor (Figure 1B) (Dyn Pick WEF-6A200-4-RCD-B; WACOH-TECH Inc., Japan). The rated capacity of the force plate in each of the mediolateral (*x*), anteroposterior (*y*), and vertical (*z*) directions was ± 10 N, whereas that of the capacitive six-axis force sensor in each of the mediolateral (*x*), anteroposterior (*y*), and vertical (*z*) directions was ± 200 N, respectively. The force plate was used for the sliding friction test under a low normal force condition (*F*_z_ ≤ 10 N), and the capacitive force sensor was used for performing the sliding friction test under a large normal force condition (*F*_z_ > 10 N). The force plate with low-rated capacity was used to accurately measure the friction force and normal force in the low normal force condition. Ten healthy young adult males (mean age: 22.3 ± 0.9 years) participated in the sliding friction test. Of these 10 participants, seven were right-handed and three were left-handed, and two of the 10 participants played baseball. The participants were informed of the study protocol, and informed consent was obtained from each participant prior to the experiment. Further, the study protocol was approved by the institutional review board of Tohoku University, Japan.

The leather skin was removed from a baseball (1BJBH43500; MIZUNO Corporation, Tokyo, Japan) and adhered to the force plate or force sensor with adhesive tape (Figure 1). The thickness of the skin was ~2 mm. Note that we aimed to measure the friction coefficient between the finger pad and a flat leather sheet (without any curvature). Each participant was instructed to slide the index finger of their dominant hand across the leather sheet. The angle made by the finger and the leather sheet while sliding was set to ~10° to keep variation in the finger angle, which may alter the contact area during sliding, to a minimum for each participant. During the low normal force-sliding test (*F*_z_ ≤ 10 N), the participants were instructed to slide their index finger in the proximal direction (the minus *y* direction in Figure 1A) 10 times with an increasing level of vertical force in a single trial, resulting in 10 different levels of vertical force within 10 N in a single trial. Conversely, during the large normal force-sliding test (*F*_z_ > 10 N), participants were instructed to slide their finger in the proximal direction (the minus *y* direction in Figure 1B) five times with an increasing level of vertical force in a single trial, i.e., resulting in five different levels of vertical force over 10 N. Here the upper limit of the normal force for each participant was the maximum value of the normal force that participants can perform, which varied among participants. Fingers were slid over ~70 mm within periods of 0.5 s, resulting in a mean sliding velocity of ~140 mm/s.

**Figure 2:**
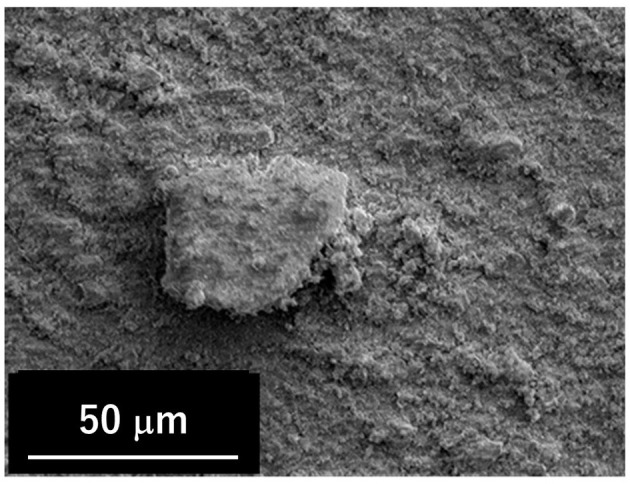
Scanning electron microscope image of rosin powder.

For each participant, there were four blocks of trials conducted for the low normal force and large normal force tests under four types of lubrication conditions, i.e., dry non-powdered condition, dry powdered condition, wet non-powdered condition, and wet powdered condition. Three trials were conducted for each lubrication condition. Under the wet non-powdered condition, purified water was sprayed five times (3 g in total) over the entire leather surface using a spray bottle. Water was also sprayed once onto the pad of the index finger. For both the dry powdered and wet powdered conditions, the participants were asked to touch a rosin bag (2ZA-416; MIZUNO Corporation, Tokyo, Japan) five times to ensure that rosin powder covered the entire pad area of the index finger. Under the wet powdered condition, rosin powder was applied in the same manner (touching a bag five times) after the finger pad was sprayed with water. Figure 2 shows a scanning electron microscope image of the rosin powder used in this study, which consisted of magnesium carbonate (80%), pine resin (15%), and petroleum resin (5%). As shown in Figure 2, magnesium carbonate particles of a few micrometers aggregated within the resin, resulting in some aggregate particles measuring dozens of micrometers in diameter.

Before each block of trials, participants were asked to wash their hands with a detergent with subsequent careful drying. Then, the moisture, oiliness, and elasticity of the finger pad were measured using a skin sensor (Triplesense® MORITEX Corporation, San Jose, CA, USA). This device consists of three sensors that can simultaneously collect and display the moisture, oiliness, and elasticity levels of the skin under examination using a scale of 0–99 points. The leather sheet was replaced with a new one (brand new one) after every trial. The participants were given a practice period to become accustomed to the demands of the experiment by sliding their index finger under different levels of normal force on the leather sheet under dry conditions at the instructed sliding speed in accordance with a dot moving linearly as the designated speed (140 mm/s) displayed on a computer monitor. The order of each block of trial was randomized to eliminate any order effect in each normal force level friction test (*F*_z_ ≤ 10 N or *F*_z_ > 10 N). The tests were conducted at 25°C ± 1°C and 50 ± 10% relative humidity.

### Data Analysis

The friction coefficient μ was calculated from normal (*F*_z_) and horizontal (*F*_y_) forces measured as follows:

(1) $$\begin{array}{l} \mu =\frac{\left|F_{y}\right|}{F_{z}} \end{array}$$

**Figure 3:**
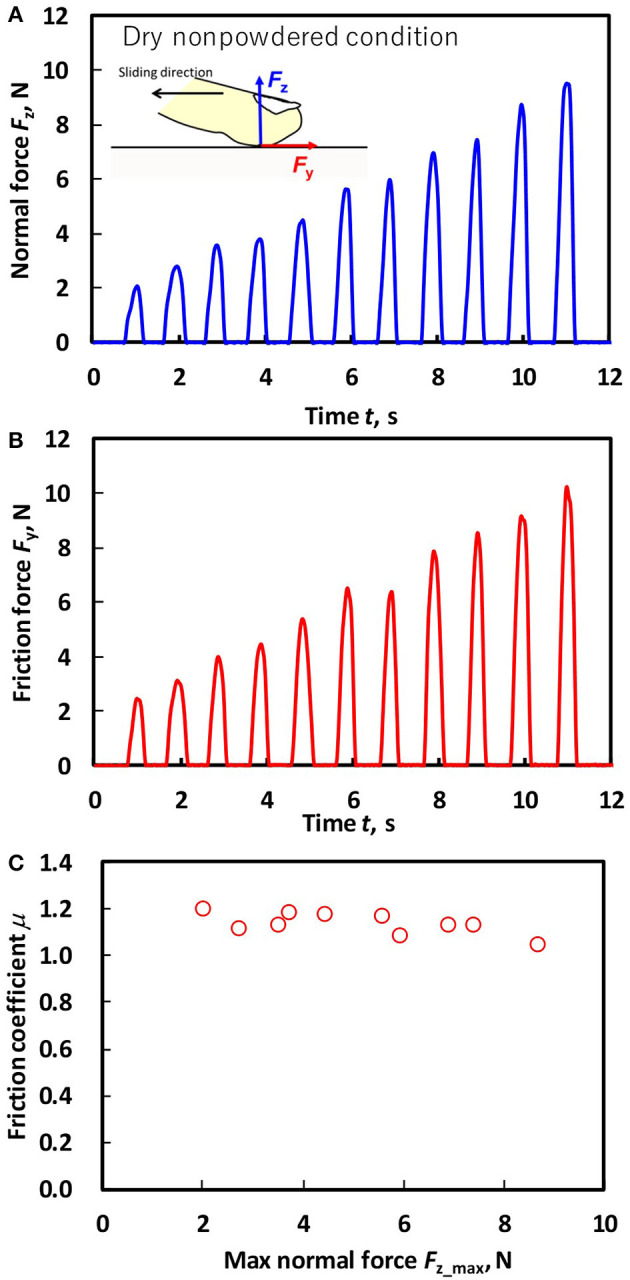
Representative time series of **(A)** the normal force (*F*_z_), **(B)** the horizontal reaction force (*F*_y_), and **(C)** the friction coefficient (μ) under the dry non-powdered condition during the low normal force condition (*F*_z_ ≤ 10 N) for participant B.

The sampling frequency of the forces was 100 Hz, and these were low pass–filtered with a cutoff frequency of 50 Hz. As shown in Figure 3, the maximum value of the normal force *F*_z_max_ in each of the 10 (in low normal force tests) or five sliding trials (in large normal force tests) and the friction coefficient at the instant of each *F*_z_max_ were used (Figure 3C) (Derler et al., 2009). The mean value of *F*_z_max_ and the corresponding friction coefficient at each normal force level for three replications were used for the analysis to investigate the effect of the normal force on the friction coefficient.

We conducted a two-way analysis of variance to investigate whether the friction coefficient was affected by the lubrication condition (dry or wet) and the application of rosin powder for each normal force condition (*F*_z_max_ ≤ 10 N and *F*_z_max_ > 10 N). A post hoc *t*-test with Bonferroni correction was used to determine specific significant differences among the four lubrication conditions. The significance level was set at *p* = 0.05.

## Results

**Figure 4:**
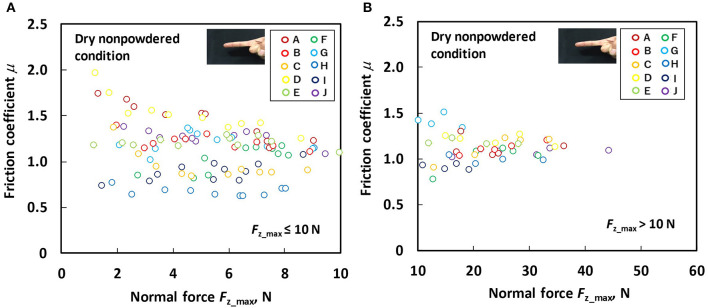
Relation between normal force (*F*_z_max_) and friction coefficient (μ) under the dry non-powdered condition **(A)** during the low normal force condition (*F*_z_max_ ≤ 10 N) and **(B)** during the large normal force condition (*F*_z_max_ > 10 N).

**Figure 5:**
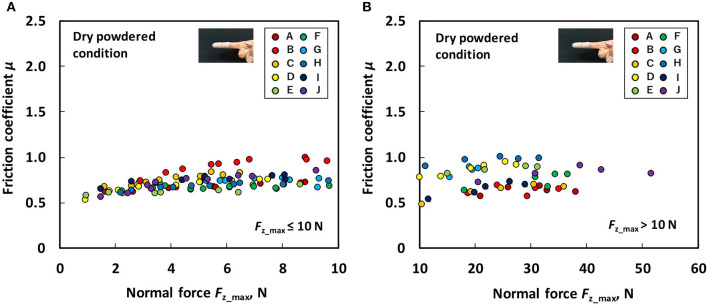
Relation between normal force (*F*_z_max_) and friction coefficient (μ) under the dry powdered condition **(A)** during the low normal force condition (*F*_z_max_ ≤ 10 N) and **(B)** during the large normal force condition (*F*_z_max_ > 10 N).

Figure 4 shows the relation between the *F*_z_max_ and friction coefficient for each participant under the dry non-powdered condition following the low normal force test (Figure 4A) and large normal force test (Figure 4B). Figure 5 shows the relation between the *F*_z_max_ and friction coefficient for each participant under the dry powdered condition following the low normal force test (Figure 5A) and large normal force test (Figure 5B). As displayed in Figures 4A, 5A, in comparison to the non-powdered condition, rosin powder application reduced the dependence of the friction coefficient on the normal force for each participant and the difference in friction coefficient among participants during low normal force-sliding testing. During large normal force-sliding tests under dry powdered condition (Figure 5B), the dependence of the friction coefficient on normal force was low, similar to that in the low normal force condition (Figure 5A).

**Figure 6:**
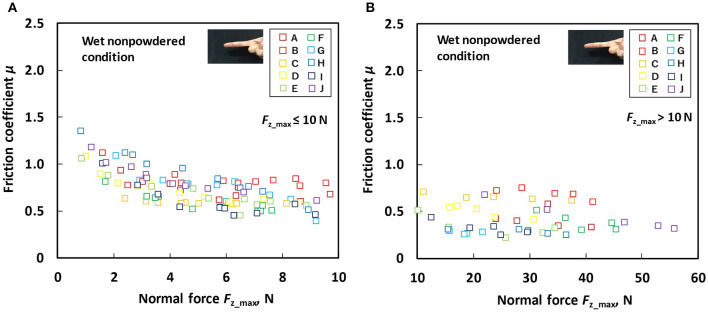
Relation between normal force (*F*_z_max_) and friction coefficient (μ) under the wet non-powdered condition **(A)** during the low normal force condition (*F*_z_max_ ≤ 10 N) and **(B)** during the large normal force condition (*F*_z_max_ > 10 N).

**Figure 7:**
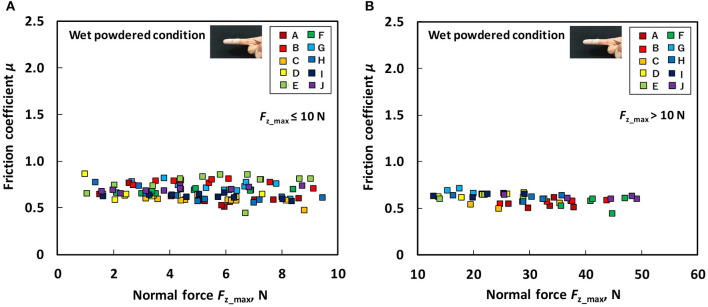
Relation between normal force (*F*_z_max_) and friction coefficient (μ) under the wet powdered condition **(A)** during low normal force condition (*F*_z_max_ ≤ 10 N) and **(B)** during large normal force condition (*F*_z_max_ > 10 N).

Figure 6 indicates the relation between the *F*_z_max_ and friction coefficient for each participant under the wet non-powdered condition at the time of the low normal force test (Figure 6A) and the large normal force test (Figure 6B). Figure 7 presents the relation between the *F*_z_max_ and friction coefficient for each participant under the wet powdered condition in the low normal force test (Figure 7A) and the large normal force test (Figure 7B). The figures indicate that, under wet conditions, powder application reduces the dependence of the friction coefficient on normal force, stabilizing the friction coefficient across a wide range of normal force conditions. The figures also demonstrate that the variation of friction among participants was also reduced by applying rosin powder under wet conditions.

**Figure 8:**
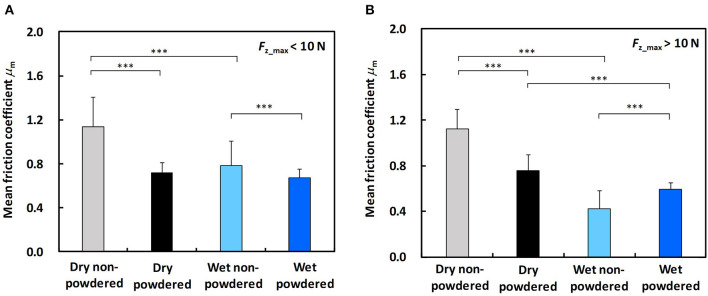
Comparison of mean and standard deviation values of the friction coefficient for the dry non-powdered, dry powdered, wet non-powdered, and wet powdered conditions **(A)** during low normal force condition (*F*_z_max_ ≤ 10 N) and **(B)** during large normal force condition (*F*_z_max_ > 10 N). ***p < 0.001.

Figure 8 shows the mean values of friction coefficients for dry non-powdered, dry powdered, wet non-powdered, and wet powdered conditions in the low (Figure 8A) and large (Figure 8B) normal force tests. As shown in Figure 8A, in the low normal force condition (*F*_z_max_ ≤ 10 N), the friction coefficient is reduced when the finger and ball were wet under non-powdered condition (*p* < 0.001), and the rosin powder application reduces the friction coefficient under dry (*p* < 0.001) and wet conditions (*p* < 0.001). In the large normal force condition (*F*_z_max_ > 10 N), the friction coefficient under the wet non-powdered condition was significantly smaller than that under the dry non-powdered condition (*p* < 0.001), and the rosin powder application reduced friction coefficient under dry (*p* < 0.001) conditions but increased the friction coefficient under wet conditions (*p* < 0.001) (Figure 8B).

**Figure 9:**
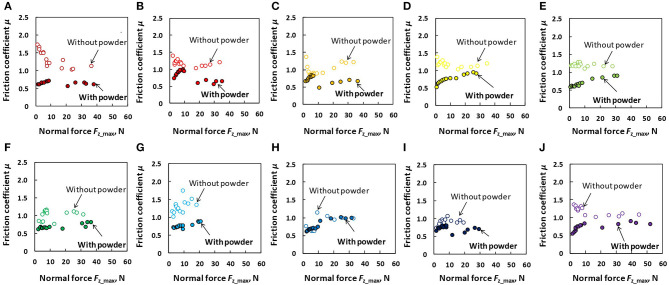
Comparison of friction coefficient (μ) as a function of normal force (*F*_z_max_) between the dry non-powdered and dry powdered conditions for each participant. **(A)** Participant A, **(B)** participant B, **(C)** participant C, **(D)** participant D, **(E)** participant E, **(F)** participant F, **(G)** participant G, **(H)** participant H, **(I)** participant I, and **(J)** participant J.

**Figure 10:**
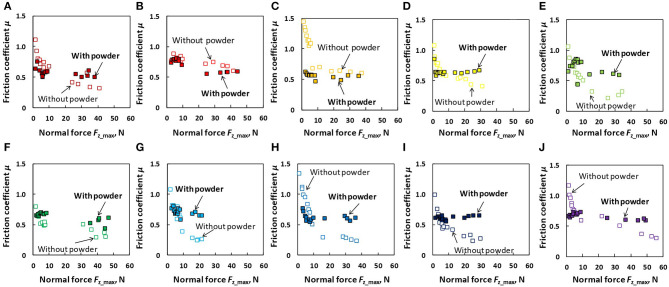
Comparison of friction coefficient (μ) as a function of normal force (*F*_z_max_) between the wet non-powdered and powdered conditions for each participant. **(A)** Participant A, **(B)** participant B, **(C)** participant C, **(D)** participant D, **(E)** participant E, **(F)** participant F, **(G)** participant G, **(H)** participant H, **(I)** participant I, and **(J)** participant J.

Figure 9 details the comparison of friction coefficient as a function of *F*_z_max_ between the dry powdered and dry non-powdered conditions for each participant. In the presented graphs, the data obtained in the low normal force and large normal force tests were merged for clarity. As shown in the figure, overall (expect participant H), the application of rosin powder on the finger pad had no effect in terms of increasing the friction coefficient under dry conditions. Figure 10 shows the comparison of the friction coefficient as a function of *F*_z_max_ between the wet powdered and non-powdered conditions for each participant. As shown in the figure, under the wet condition, rosin powder application is effective in increasing the friction between the finger pad and baseball surface in larger normal force conditions, except for participants B and C; however, at low normal force conditions, rosin powder application instead decreases the friction compared with that in the non-powdered condition.

## Discussion

Our results indicated that, under dry conditions, the friction coefficient decreased when rosin powder was applied on the finger pad compared with under the non-powdered condition over a wide range of normal force values, but rosin powder application reduced the variation in friction coefficient among participants at low normal force conditions compared with the non-powdered condition (Figures 4A, 5A). One possible reason for the large variation in the friction coefficient under the dry non-powdered condition during low normal force testing was the difference in the amount of moisture (hydration) in the finger pad skin among the participants, which would have influenced adhesion between the finger pad and the contacting surface (Adams et al., 2007; Derler et al., 2007, 2009; André et al., 2011; Pasumarty et al., 2011; Tomlinson et al., 2011; Derler and Gerhardt, 2012). The moist skin becomes softer and is characterized by a lower elastic modulus so that adhesion is increased (Wolfram, 1983). This was supported by the fact that the moisture level of the finger of each participant measured using the skin sensor before testing tended to indicate a positive correlation with the mean friction coefficient under the low normal force condition (*F*_z_max_ ≤ 10 N) (Pearson's product moment correlation coefficient, *r* = 0.530; significance, *p* = 0.11). The oiliness and elasticity levels showed no significant correlation with the friction coefficient at this normal force range. The reduction in the variation of the friction coefficient among participants for rosin-powdered conditions (Figure 5A) is possibly correlated with the occurrence of shear mainly within the rosin powder layers, which would reduce the effect of skin conditions such as moisture content on adhesion friction. The variation of the friction coefficient among participants was smaller in the wet non-powdered (Figure 6A) and wet powdered conditions (Figure 7A) than in the dry non-powdered condition (Figure 4A) at low normal force, which could also be a result of the inhibition of the difference in the skin conditions, such as hydration among participants, because the finger was covered with the rosin powder, water film, or both. The reduction in the magnitude of friction coefficient under the dry powdered condition compared with the dry non-powdered condition was possibly a result of the reduced contact area between the finger and leather as well as a reduction in shear strength at the interface, acting as a solid lubricant (Carré et al., 2012) and reducing adhesion friction (Spinner et al., 2016).

The results obtained from the wet conditions indicated that the friction coefficient under the wet non-powdered condition tends to decrease with increasing normal force (Figure 6), whereas rosin powder application had the effect of suppressing the reduction in the friction coefficient with respect to normal force, stabilizing the friction over a wide range of normal force (Figure 7). The friction coefficient μ can be expressed as a function of normal force *F*_z_ as follows (Derler et al., 2009):

(2) $$\begin{array}{l} \mu ={kF}_{\text{z}}^{\text{n}-1} \end{array}$$

where *k* is constant and *n* is the load index. In the logarithmic form of Equation 2, the exponent *n–*1 was determined by linear regression to test whether the measured friction coefficient data under the wet non-powdered condition can be attributed to a certain predominant friction mechanism. Friction mechanism such as adhesion, deformation (hysteresis), or hydrodynamic lubrication should be indicated by distinctive exponents n – 1 of –1/3, 1/3, and –1, respectively (Derler et al., 2009). The exponent for our wet non-powdered condition data shown in Figure 10 was –0.09 to –0.69 (mean ± standard deviation: –0.36 ± 0.17). This means that the predominant friction mechanism for the wet non-powdered condition could be adhesion or mixed lubrication, i.e., a coexistence of adhesion and hydrodynamic lubrication for most participants. Therefore, it can be assumed that water films at the contact area between the finger pad and baseball are formed locally, whereas the dry contact area coexists in the other area. Conversely, when rosin powder was applied, the effect of water lubrication could be neglected due to absorption of the water film by the rosin powder, resulting in stable friction with respect to normal force. As a result, rosin powder application increases friction under large normal force compared with no powder application. The available literature indicates that the normal force applied at the index finger and middle finger exhibits a bimodal pattern with initial and second peaks at 40 and 6–7 ms before ball release, respectively (Kinoshita et al., 2017). After the second peak of the normal force, the normal force reduces to 0 N at the instant of ball release. When considering the abovementioned normal force variation during the ball-releasing process in pitching, the rosin powder acts as a grip-enhancing agent in the face of a large normal force condition, i.e., *F*_z_ > 10 N, meaning that the powder does not act as a grip-enhancing agent during the whole ball-releasing process and may even cause slippage at the very end of ball release, at which point the normal force of the finger is low, e.g., *F*_z_ ≤ 10 N.

A large friction coefficient between the finger pad and the ball can increase the maximum shear force that can be applied to the ball surface in the tangential direction. The shear force applied at the point of contact between the finger and the ball is thought to increase the ball spin rate in actual pitching (to the best of our knowledge, however, there is no literature supporting this relationship in actual pitching). In addition, other studies (Jinji et al., 2008; Nagami et al., 2011) indicate that the ball spin rate is highly correlated with the ball velocity in fastball pitching, which could be a result of the increase in resultant force applied to the ball. In the dry conditions, our results indicated that the friction coefficient was not necessarily increased by rosin powder application, which could not have increased the spin rate and fastball velocity. However, in the wet conditions, in addition to the efficacy in preventing ball slips during the ball-releasing process, the rosin powder application could increase shear force, except just before the ball is released (corresponding to the low normal force condition), which may result in the increases in ball spin rate and fastball velocity. However, future work is needed to confirm these effects of rosin powder application through actual pitching trials in different finger-ball lubrication conditions.

In both dry and wet conditions, we found that rosin powder application stabilizes the friction coefficient with respect to the change in normal force. The fact that the friction coefficient was not significantly affected by normal force indicates that the friction force between a finger pad and baseball surface is proportional to the normal force, i.e., Amontons' 1st law (Bowden and Tabor, 1950). This linear relation between the normal force and friction force may facilitate control of the friction force between a finger pad and baseball: in other words, the friction force for preventing slips and increasing the shear force for the ball spin rate can be linearly controlled by the normal force, i.e., grip force applied to the ball by fingers. In contrast, under dry and wet non-powdered conditions, the relationship between friction force (shear force) and normal force (grip force) was non-linear, which could have caused some difficulty in controlling the slip and spin rates of the ball by grip force.

The difference in the mean friction coefficient between the dry and wet non-powdered conditions was 0.351 for the low normal force condition (*F*_z_max_ ≤ 10 N) and 0.702 for the large normal force condition (*F*_z_max_ > 10 N). Conversely, the difference in the mean friction coefficient between the dry and wet powdered conditions was 0.051 for the low normal force condition (*F*_z_max_ ≤ 10 N) and 0.167 for the large normal force condition (*F*_z_max_ > 10 N). This demonstrates that rosin powder application inhibits the reduction in the friction coefficient due to the existence of water at the interface, which could be due to the absorption of the water film existing on the finger pad and ball surface by rosin powder. This diminishes the effect of the lubricating water film, and the shear occurs mainly within the rosin powder layers even under wet conditions, which enables a similar frictional property to that seen under the dry powdered condition. Therefore, the usage of rosin powder may have an advantage here in maintaining the frictional property between finger and baseball as stable, regardless of skin surface condition such as dry and wet conditions.

There are some noteworthy limitations to this study. The leather skin of a brand new ball was used for every trial; a ball treated by rubbing mud was not used. The presence of mud on the ball's leather skin would affect the friction between finger and ball even when the rosin powder is applied to the finger pad surface. Thus, a further study is necessary to investigate this effect. The maximum value of the normal force applied between a finger pad and baseball surface in the sliding friction test (*F*_z_max_ <60 N) may be lower than that during fastball pitching (Kinoshita et al., 2017). The normal force applied at the fingers increases with increasing ball speed (Kinoshita et al., 2017); thus, it is predicted from the results obtained in the wet sliding test that the difference between friction coefficients under powdered and non-powdered conditions may increase under the wet condition; thus, the importance of rosin powder application is expected to increase in fastball pitching when the ball and finger are wet. Another limitation of this study is the fact that the finger pad was in contact with the flat leather sheet of the baseball rather than the curved surface and seam of the ball. The contact between a finger and the curved ball and seam should increase the contact pressure and affect the friction behavior, which should be investigated in the future. Most pitching types involve the use of two fingers, the index and middle finger, to grip a baseball. However, only an index finger was tested in this study. The angle made by the finger and the leather sheet while sliding (~10°) and the sliding velocity were not arranged to simulate real pitching. These situations also limited our results. Our results were not obtained through an actual pitching trial; thus, further research is needed to investigate the effect of rosin powder application on friction between fingers and the ball, as well as on pitching performance, through an actual pitching test.

## Conclusions

To our knowledge, the present study is the first attempt to investigate the effect of rosin powder application on the sliding friction behavior between a finger pad and a baseball leather sheet. Our findings suggest that rosin powder application stabilizes friction under both dry and wet conditions, with less dependency of the friction coefficient on the normal force and less variation in the friction coefficient among individuals. Therefore, rosin powder application could maintain friction at the same level in different environmental conditions, such as dry and wet conditions, and could linearly increase friction force (shear force) between the finger pad and ball in real pitching by increasing the grip force. For most participants, the friction coefficient was not necessarily increased by the presence of rosin powder at the finger pad–leather sheet interface under dry conditions. However, under wet conditions, rosin powder application increased the friction coefficient in the large normal force condition compared with that in the non-powdered condition, indicating the efficacy of rosin powder application as a grip-enhancing agent in the normal force range when the ball and finger are wet. Rosin powder application may also be able to increase shear force applied to the ball during the ball-releasing process, which may result in an increase in the ball spin rate and fastball velocity. Our results provide new insights into the role of rosin powder in altering the frictional behavior between a finger and a baseball during pitching. Further study is needed to investigate how rosin powder application affects the frictional property between fingers and ball during pitching and pitching performance such as ball velocity and ball spin rate.

## Data Availability Statement

The datasets generated for this study will be made available by the authors, after explicit and justified request, to any qualified researcher.

## Ethics Statement

The studies involving human participants were reviewed and approved by the institutional review board of Tohoku University. The patients/participants provided their written informed consent to participate in this study. Written informed consent was obtained from the individual(s) for the publication of any potentially identifiable images or data included in this article.

## Author Contributions

TY, TF, and DN contributed to the design of the study. TY, NY, and SM acquired the data and performed the data analysis. Drafting of the manuscript was performed by TY and DN. All authors contributed to interpretation of the data, participated in revising the manuscript, and approving the final submission.

### Conflict of Interest

TF and DN are employed by the company Nippon Telegraph and Telephone Corporation. The remaining authors declare that the research was conducted in the absence of any commercial or financial relationships that could be construed as a potential conflict of interest.
